# Short-Term Characterization of Spherical 100Cr6 Steel Samples Using Micro Compression Test

**DOI:** 10.3390/ma13030733

**Published:** 2020-02-06

**Authors:** Heike Sonnenberg, Brigitte Clausen

**Affiliations:** 1 Faculty of Production Engineering, University of Bremen, Badgasteiner Straße 1, 28359 Bremen, Germany; clausen@iwt-bremen.de; 2 Leibniz Institute for Materials Engineering–IWT, Badgasteiner Straße 3, 28359 Bremen, Germany

**Keywords:** compression test, spherical micro samples, mechanical descriptor, force–displacement curves, bearing steel 100Cr6, microstructure

## Abstract

For the establishment of a novel development process of new structural materials, short-term characterization methods capable of testing hundreds of spherical micro samples are needed. This paper introduces a compression test on spherical micro samples as a short-term characterization method to investigate the elastic-plastic deformation behavior. To demonstrate the potential of this newly developed method, the micro compression test is performed with a maximum loading of 300 N on 100Cr6 (AISI 52100 bearing steel) samples, with a diameter of 0.8 mm, in 15 different heat treatment conditions. The austenitizing temperature is varied between 800 and 1150 °C. Tempering of the samples is carried out in a differential scanning calorimetry process with temperatures of 180, 230 and 300 °C. Out of force-displacement curves and stress-strain relations, so-called descriptors (characteristic values) which are sensitive to the applied heat treatment can be extracted. The change of mechanical properties due to heat treatment and the resulting microstructure is presented by the trend of a stress descriptor in dependence of austenitizing and annealing temperature, which can be compared to the trend of the tensile strength as a material property obtained by conventional tensile tests. The trend of the descriptor determined in the compression test on spherical samples indicates the validity of this approach as a short-term characterization method.

## 1. Introduction

Compression tests on spherical micro samples are usually performed as single-grain or single-particle experiments or experiments on agglomerates for the characterization of particles in powders, granules or medical products. There are several publications about compression tests on small particles in which the complex state of stress as well as crack and breakage behavior due to compression are investigated [1,2,3,4,5,6,7,8,9,10,11,12,13,14]. Particle fracture and breakage behavior [1,2,3,4,5,6], the development of stress and strain during compression of spheres [2,7,8,9] and energy absorption at different strain rates [10] are often described in finite element models and are underlined with experimental investigations [1,2,3,4,5,8,10]. The experimental particle compression testing is performed with either static or cyclic loading. The main challenge due to geometrical boundary constraints (i.e., the contact problem of sphere and plane and the influence of stiffness and rigidity of the contact partners) is well described. The most relevant influences on particle breakage behavior is related to the material and conditions of the contact partners [5,9], particle size, form (roundness) and porosity of the particles due to manufacturing processes [6,7,12]. Within the experimental investigations, different materials and particle sizes are evaluated (e.g., granules like quartz, aluminum oxide, silica, zirconium and sodium chloride or acrylic glass), but no structural materials like steel alloys. The particle diameters in these studies range from exemplary 20 to 60 µm [9], 500 to 700 µm [6], 1 to 4 mm [11] and up to 50 mm [7], with varying loading forces from 1 to 30 N. Nevertheless, the aim of those investigations usually is the examination of a particle as one constituent in bulk material and the interactions of those particles during transportation, processing and storage (e.g., impact stress and abrasion).

A material characterization by particle crushing is possible as it is shown for sand particles. The mechanical behavior of quartz can be described even for particles with irregular morphology in a diameter range of 1 to 4 mm [11]. Additionally, a finite element model is developed for the characterization of the hoop strength assuming linear elastic and brittle material behavior of a compressed spherical sample [3]. The prediction of the material behavior of a metal particle during compression is more complex, since non-linear elasticity can occur as well as plasticity caused by motion of dislocations, stress-induced phase transformation and twinning. Phase-field models are developed for crystalline materials and focus on mechanisms during elastic and plastic deformation. Those phase-field models are used to describe material behavior and properties (e.g., stress-induced martensitic phase transformation) [13]. One investigated mechanism is, for instance, multiple twinning, which occurs for low temperatures, high strain rates and small grains, and is evaluated on a nanoscale [14]. For a comparison of the existing models and simulations and the published experimental investigations of compression tests, the difference in evaluated length scale needs to be considered, since the technological and geometrical size effect is a challenge in mechanical testing and material characterization. However, the focus of this study is on the development of a test method to perform a mechanical material characterization for structural materials, like steel alloys, by compression of spherical micro samples.

**Figure 1:**
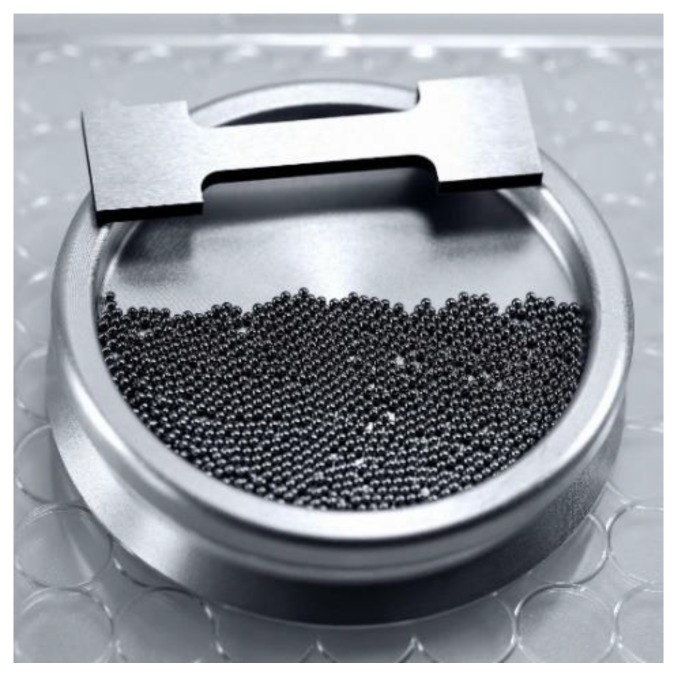
Quantity advantage: A total of 1315 microspheres with the same mass (5.37 g) as one flat tensile specimen (© Jan Rathke) [16].

For the establishment of a novel development process of new structural materials within the Collaborative Research Center “Farbige Zustände” (CRC 1232), micro samples are used for a cost- and resource-efficient material variation [15,16]. The mass of a conventional flat tensile specimen (5.37 g) is equal to the mass of 1315 spherical micro samples (Figure 1). The spherical micro samples are provided by single droplet generators, as described in [17], which enable a fast manufacturing of a high number of samples. A compression test on spherical micro samples is introduced to investigate the elastic-plastic deformation behavior. So-called descriptors (characteristic values) are delivered to enable a comparison of values of short-term characterization techniques to conventional material properties. The descriptor-based high-throughput approach of material characterization of the CRC 1232 is explained in detail in [18].

**Figure 2:**
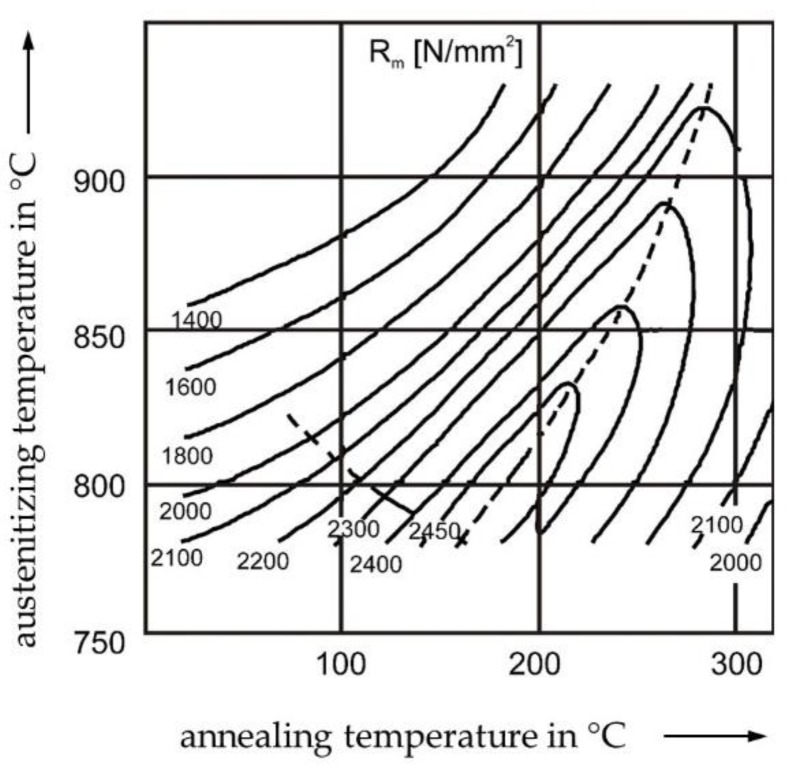
Lines of equal tensile strength *R*_m_ determined in conventional tensile tests in dependence of austenitizing and annealing temperatures according to [19].

Material properties like strength, ductility and hardness are a result of the microstructure of the material. The complex changes of the tensile strength *R*_m_ of conventional tensile tests due to heat treatment is shown in Figure 2 using 100Cr6 as an exemplary alloy in the Fe-C-Cr system. Due to the superposition of different effects on the microstructure during heat treatment, the development of the tensile strength in dependence of austenitizing and annealing temperatures is resulting in the displayed trend [19].

Micro samples can not only be generated very fast but are also easily further processed in terms of heat treatment using a differential scanning calorimetry (DSC) process. Calorimetry offers precise temperature adjustment possibilities so that tempering in the DSC process of previously hardened microspheres is an adequate heat treatment facility regarding ecological sustainability.

The main aim of this paper is to introduce the micro compression test method as a descriptor providing short-term characterization method within the CRC 1232 to improve and simplify the development of new structural materials. Results of this newly developed micro compression test are compared to the tensile strength of conventional tensile tests.

## 2. Materials and Methods

### 2.1. Materials

For the evaluation of the micro compression test method, conventionally manufactured bearing balls with diameters of 0.8 mm were used as spherical micro samples. The bearing balls consist of AISI 52100 (German grade 100Cr6). This material was used for two reasons: First, the heat treatment properties of 100Cr6 are excellent for adjusting a wide range of microstructural conditions and thus different mechanical properties. Second, the material is available as microspheres in similar sizes as the droplets generated in the future high-throughput method “Farbige Zustände” within the CRC 1232. The tolerance in terms of geometric accuracy of the available microspheres is less than 3%, making it perfect for the validation of the new micro compression test on spherical samples since particle size and form are the main influences on deformation behavior [6,8,9]. The chemical composition of the investigated samples as well as the allowed range of 100Cr6 according to DIN EN ISO 683-17:2000-04 [20] are presented in Table 1.

**Figure 3:**
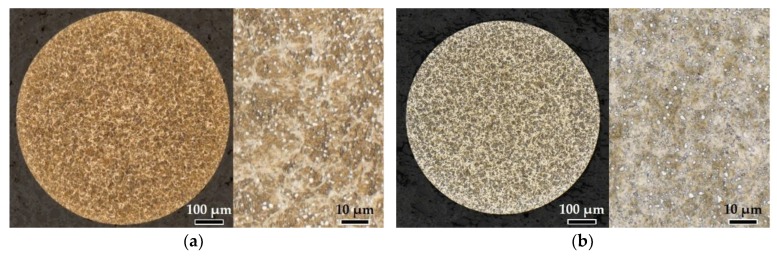

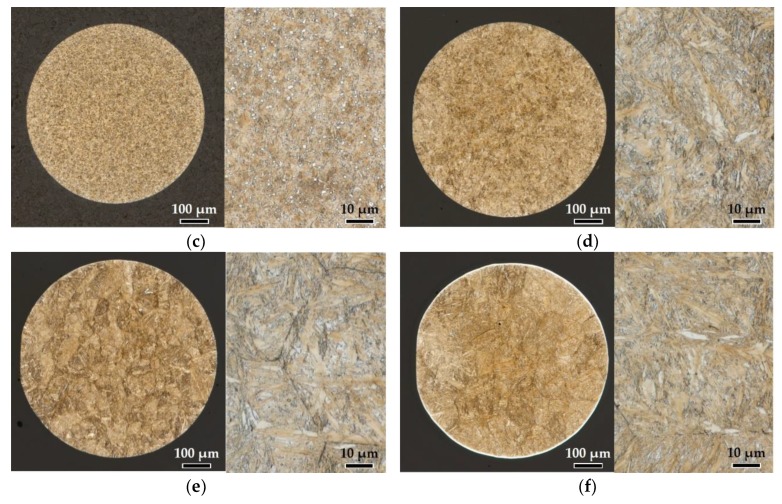
Metallographic microsection images in the equatorial plane of the micro samples in initial hardening conditions before DSC treatment: (**a**) Condition as bought; (**b**) Q1 with *T*_A_ = 800 °C; (**c**) Q2 with *T*_A_ = 850 °C; (**d**) Q3 with *T*_A_ = 950 °C; (**e**) Q4 with *T*_A_ = 1050 °C; (**f**) Q5 with *T*_A_ = 1150 °C.

The heat treatment of the microspheres was varied. The samples were heated to a defined austenitizing temperature *T*_A_ with a heating rate of 15 K/min in a vacuum furnace. Five different initial hardening conditions (Q1–Q5) with varying austenitizing temperatures of 800 °C (Q1), 850 °C (Q2), 950 °C (Q3), 1050 °C (Q4) and 1150 °C (Q5) were investigated. During heat treatment, a special sample rack with small compartments (5 × 5 × 1.5 mm) with micro wire gauze top and bottom enclosure, developed for vacuum heat treatment with subsequent high pressure gas quenching, was utilized. The perforated plate was used as a thermal buffer while the wire gauze ensures a high quenching rate by allowing the quenching gas to pass through. The micro samples were stored in sets of 20 in different compartments in order to ensure equal temperature distribution. For achieving a homogeneous carbon distribution within austenite, the holding time at austenitizing temperature was set to one hour. After austenitizing, the samples were directly quenched with nitrogen at 8 bar for the conditions Q3, Q4 and Q5, and 10 bar for Q2. The only exception in the heat treatment process was made for the condition Q1 (*T*_A_ = 800 °C). Here, heating was performed in a wire gauze enclosure in molten salt (GS540 at *T*_A_ = 800 °C) and a subsequent cooling in non-agitated water to ensure martensite transformation by reaching a sufficient quenching rate. The effect of heat treatment on the microstructure is shown in Figure 3 by exemplary microsection images.

The initial microstructure of the microspheres shows a martensitic structure with homogenously distributed carbides (white dots) displayed in Figure 3a. After the applied heat treatments with hardening at low austenitizing temperatures of 800 °C (Q1, Figure 3b) and 850 °C (Q2, Figure 3c) the undissolved, mostly spherical carbides remained in a very fine martensitic microstructure. The amount of carbides decreased with increasing austenitizing temperatures, so that in the metallographic microsection image of the heat treatment condition Q3 (*T*_A_ = 950 °C, Figure 3d) only a few carbides were still visible. Additionally, the increase of the austenitizing temperature lead to grain growth and remaining retained austenite. Compared to the other hardened initial conditions, an increase of grain size could be determined for Q3 to Q5 (Figure 3d–f).

After hardening, the micro samples were tempered with a differential scanning calorimeter (DSC). For the DSC experiments, a calorimeter of the type HT TGA/DSC 3+ (© METTLER TOLEDO, Hamburg, Germany) was used. The heating rate was set to 10 K/min with a holding time at annealing temperature of 120 min and a cooling rate of 50 K/min. Three annealing temperatures *T*_T_ (180, 230 and 300 °C) for each initial hardening condition were investigated resulting in 15 different heat treatment conditions. In the following, the 15 conditions were referred to by the abbreviated description of the austenitizing temperature (Q1–Q5) and the annealing temperature (e.g., Q1-180 °C).

### 2.2. Compression Test on Spherical Micro Samples

An instrumented micro compression test method is introduced for the mechanical testing of the spherical samples. A universal hardness testing device of the type ZHU2.5 (Zwick/Roell©, Ulm, Germany) with a modified flat compression die was used for the experiments. The maximum possible testing load of this device is 2.5 kN. The continuously measured force and displacement enabled the determination of the elastic-plastic deformation behavior of the microsphere in the loading and the unloading phase during compression. The displacement resolution of the used measurement equipment is 0.02 µm. The quasi-static compression tests were carried out with a force-controlled loading with a load velocity of 10 N/s and a displacement-controlled unloading of 0.1 mm/min. When the maximum testing force was reached, a holding time of 2 s was set. The applied maximum testing force in this investigation was set to *F*_max_ = 300 N.

**Figure 4:**
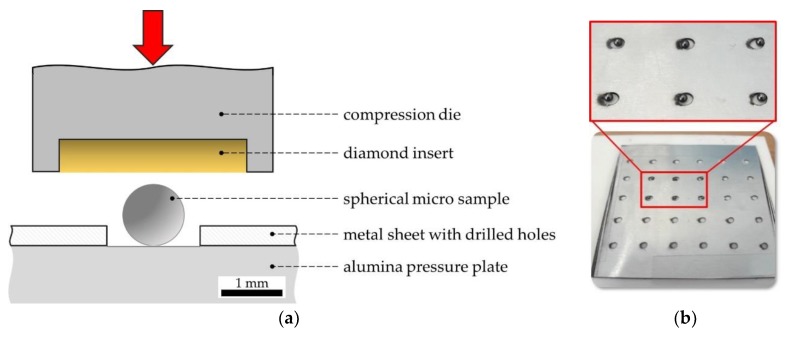
Experimental test setup of the compression test on spherical micro samples: (**a**) Sketch and labels; (**b**) photo of the positioning setup.

The spherical micro samples were manually positioned on the lower pressure plate, which was made of alumina with a compressive strength of about 4000 MPa, and were loaded one at a time by the vertical movable compression die with a diamond insert of a diameter of 3 mm (Figure 4). To ensure the separation and the individual testing of the spheres, as well as to avoid the loss of samples due to geometrical issues, an adequately thin metal sheet with drilled holes of 1.3 mm in diameter was used. Further fixation of the samples (e.g., adhesive attachment) did not take place in order to minimize influences on the measured force–displacement curves during compressive deformation.

For statistical purposes, ten experiments of each material condition were performed. Thus, all in all, 150 compression tests were carried out, each providing continuously measured force–displacement curves for a loading of 300 N. In the following, all displayed figures containing plots of descriptors show the arithmetic mean ($\bar{x}$) calculated via

(1) $$\bar{x}=\frac{1}{n}\overset{n}{\underset{i=1}{\sum }}x_{i}$$

where *n* is the sample size with *n* = 10. The relating standard deviation ($\bar{s}$) is used to evaluate the scattering of the data and is determined with Equation (2).

(2) $$\bar{s}=\sqrt{\frac{1}{n}\overset{n}{\underset{i=1}{\sum }}{\left(x_{i}-\bar{x}\right)}^{2}}$$

## 3. Results

### 3.1. Extracting Descriptors from Data of Micro Compression Tests

**Figure 5:**
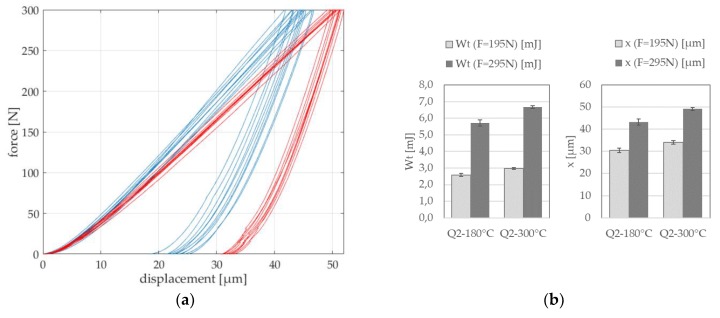
Extracting descriptors from micro compression tests on spherical samples: (**a**) Force-displacement curves of heat treatment conditions Q2-180 °C (blue) and Q2-300 °C (red); (**b**) exemplary evaluation of the mechanical work *W*_t_ and the displacement *x* each at loads of 195 and 295 N.

Force–displacement curves of instrumented micro compression tests can be directly compared if the sample size and the applied loading are not changing within the experimental investigation. The measured data of the loading phase during compression testing includes elastic and plastic deformation behavior due to the spherical sample geometry. Extracted mechanical descriptors from force–displacement curves, for example, are the mechanical work *W*_t_ (area under curve), the displacement *x* at a certain load as well as slopes of the curve. Exemplarily, the experimental results of two heat treatment conditions Q2-180 °C (blue) and Q2-300 °C (red) are shown in Figure 5. The measured force–displacement curves of ten samples of each heat treatment condition and their scattering for a maximum load of 300 N can be seen. The examined variations are small and the trends of the curves are similar for each heat treatment. However, differences between the two heat treatment conditions can be obtained (Figure 5a). The arithmetic mean value and the standard deviation (according to Equations (1) and (2)) of the mechanical work *W*_t_ (in mJ) and the total displacement *x* (in µm), as descriptors of the micro compression test, are exemplarily evaluated in Figure 5b for the loading phase at loads of 195 and 295 N.

**Figure 6:**
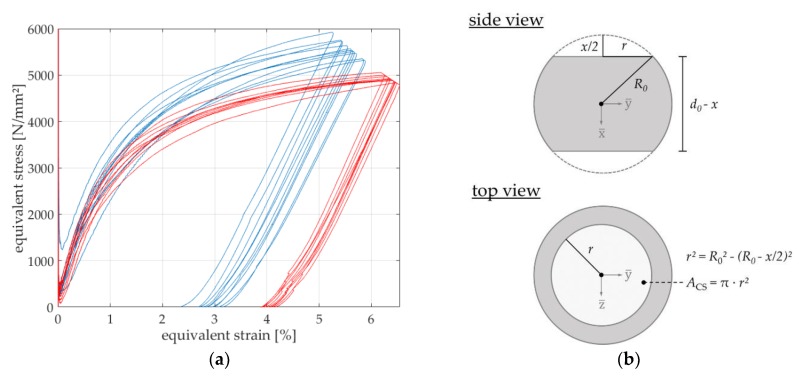
(**a**) Results of micro compression tests (equivalent stress-strain relation) of the heat treatment conditions Q2-180 °C (blue) and Q2-300 °C (red); (**b**) determination of the area of the circle segment $A_{CS}$ for the calculation of σ according to Equation (3) of a theoretically purely plastically deformed microsphere after compression.

Geometrical effects should not affect the resulting descriptor. For normalizing the experimental measured data, a stress equivalent was used. Thus, in analogy to stress-strain-diagrams of tensile tests, the influence of a different initial sample diameter or a change of the applied testing force is (in certain domains) excluded. As shown in [21], there might be a technological size effect regardless of the geometrical effect. The stress equivalent σ, which is displayed on the ordinate axis (Figure 6a), is based on the formula

(3) $$\sigma =\frac{F}{A_{CS}}$$

in N/mm^2^, and can be evaluated during the whole compression since the force F and the displacement x are continuously measured. The reference area $A_{CS}$

(4) $$A_{CS}=\pi *r^{2}$$

is the area of a circle segment as a function of the current displacement *x*, assuming pure plastic deformation behavior (Figure 6b). This assumption includes the hypothesis that the difference in deformation of the sample due to different contact partners (alumina and diamond) on both contact sides is negligible. Based on the geometrical relations according to Pythagoras, the radius r of the current reference area is determined with

(5) $$r^{2}=R_{0}^{2}-{\left(R_{0}-\frac{x}{2}\right)}^{2}$$

in which $R_{0}$ is the initial radius of the microsphere (Figure 6b). The strain ε is displayed at the x-coordinate axis and takes the initial sphere diameter $d_{0}$ into account. It is; therefore, calculated via

(6) $$\varepsilon =\frac{x}{d_{0}}*100$$

and evaluated in %. ε is displayed as absolute value even though, of course, the elongation is negative during micro compression testing.

Due to point contact in the beginning of the compression test on spherical micro samples, maximum stress values occur since the reference area tends to zero (Figure 6b). Those values are not considered as global maximum stress values since they occur due to plastic deformation of the initial contact zone (point contact). Therefore, only data of a strain above 0.1% are defined as valid when determining maximum values in automated data process routines. Thus, similar to the extraction of descriptors out of force–displacement curves, stress–strain diagrams can be evaluated. Exemplarily, the equivalent stresses at strains of ε=3.5% and ε=5% of the loading phase can be extracted and are used as normalized descriptors from micro compression tests on spherical samples.

### 3.2. Descriptors from Micro Compression Tests after Heat Treatment by Differential Scanning Calorimetry

**Figure 7:**
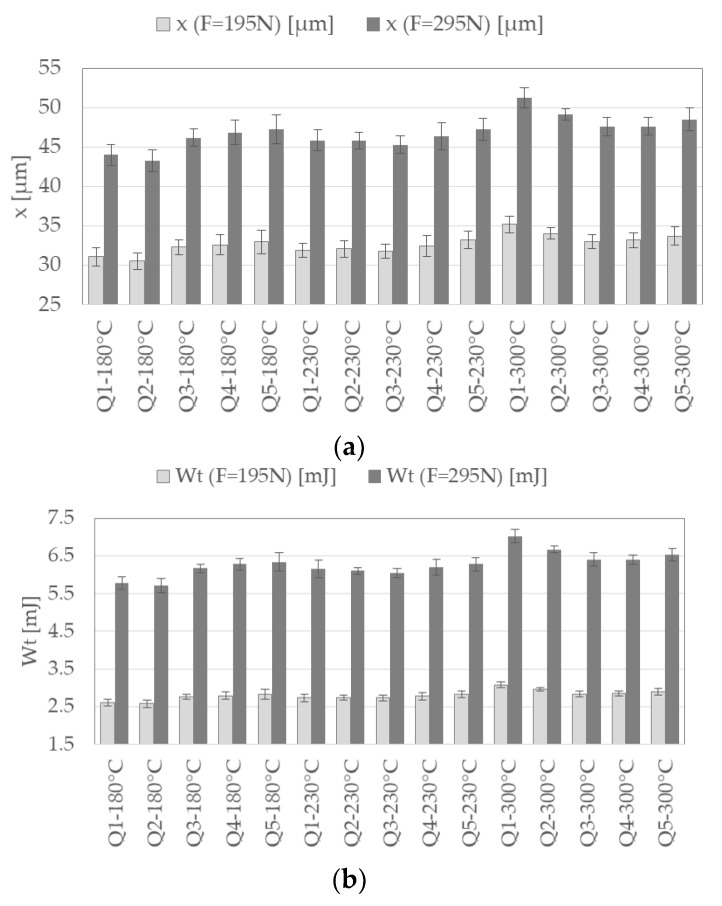
Descriptors of micro compression tests of the investigated heat treatment conditions of 100Cr6 extracted from force–displacement curves and ordered by annealing temperature: (**a**) Displacement *x* at a loading of F = 195 N and F = 295 N; (**b**) mechanical work *W*_t_ at a loading of F = 195 N and F = 295 N.

In Figure 7, an overview on the extracted descriptors from force-displacement curves of micro compression tests of all investigated heat treatment conditions of 100Cr6 is presented. The results are ordered by annealing temperature *T*_T_ of the heat treatment during DSC. The displayed descriptors are the displacement *x* due to compression (Figure 7a) and the mechanical work *W*_t_ (Figure 7b), each at a load of F = 195 N and F = 295 N during the loading phase. Arithmetic mean values and standard deviations (according to Equations (1) and (2)) for ten measurements each are shown. For every selected descriptor, differences in the investigated heat treatment condition of 100Cr6 can be seen and standard deviations are small. With increasing austenitizing temperature (Q1–Q5), the obtained values for the displacement as well as the mechanical work first decrease to a minimum and then increase again. For the different annealing temperatures the minimum value occurs at different austenitizing temperatures. When comparing the descriptors of the two load levels the overall trends are similar. However, the trend of descriptors extracted from higher load levels tend to be more pronounced.

**Figure 8:**
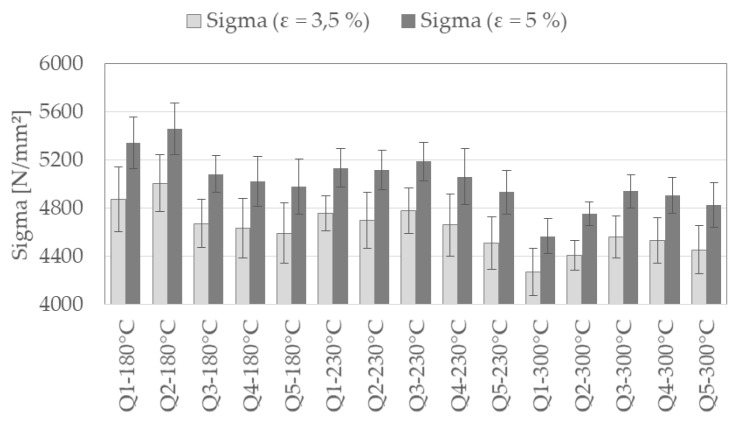
Stress descriptor, σ, at two different strains (3.5% and 5%) extracted from stress-strain relations of micro compression tests of the investigated heat treatment conditions of 100Cr6, ordered by annealing temperature.

Furthermore, the equivalent stress σ, as a descriptor out of calculated stress-strain relations based on the contact surface area described in Section 3.1, is evaluated. This stress, σ, is extracted for two different strains, ε, of 3.5% and 5% and is presented in Figure 8. The results (arithmetic mean and standard deviations according to Equations (1) and (2)) are also ordered by heat treatment condition during DSC (annealing temperature *T*_T_). Like the trends of descriptors of force-displacement curves, differences in stress descriptor values between the investigated material conditions of 100Cr6 can be seen. The stress values are first increasing and, after a maximum value, decreasing with austenitizing temperature. For the different annealing temperatures the maximum value occurs at different austenitizing temperatures. The standard deviations of the descriptors obtained at lower strains (ε=3.5%) are bigger than for higher strain levels.

## 4. Discussion

**Figure 9:**
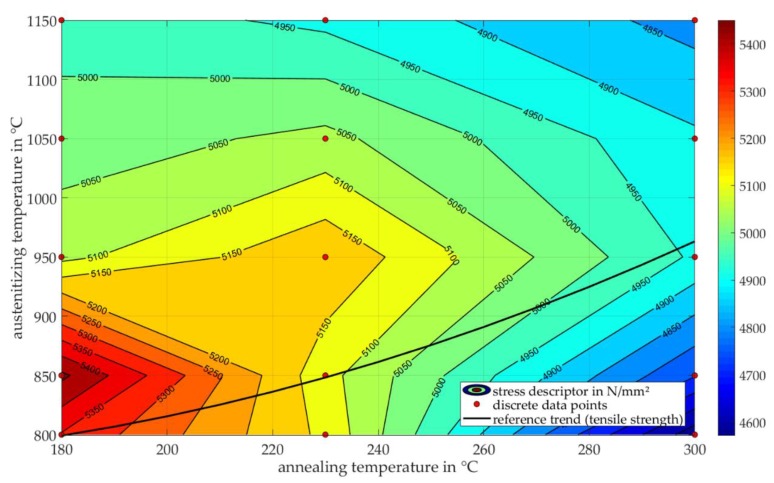
Contour plot (*T*_A_ over *T*_T_) of experimentally determined equivalent stress values $\sigma_{\left(\varepsilon =5\%\right)}$ at strains of 5% in N/mm^2^ as a descriptor of micro compression tests on spherical 100Cr6 samples, with investigated data points (red dots) and a comparative reference line (black) for the possibility of transferability to the trend of tensile strength of conventional tensile tests [19].

All the presented descriptors, which can be determined by the newly developed micro compression testing method of spherical samples, show a significant sensitivity in dependence of the heat treatment condition. However, the visualization of the obtained data can be improved. Inspired by the contour plot of the tensile strength *R*_m_ in dependence of austenitizing temperature and annealing temperature (Figure 2), the stress descriptor, σ, at a strain of ε=5% is used to create an analog interpolated graphic (Figure 9) for the investigated annealing temperatures *T*_T_ = 180–360 °C and austenitizing temperatures of *T*_A_ = 800–1150 °C, revealing a much more informative visualization of the data. The contour plot of $\sigma_{\left(\varepsilon =5\%\right)}$ of the micro compression test is generated by interpolating the 15 discrete data points (arithmetic mean values) of the examined heat treatment conditions, which are marked as red dots in Figure 9.

With the help of this contour plot, the effects of heat treatment on the mechanical response of the material condition can be classified and evaluated. First of all, there is a maximum of $\sigma_{\left(\varepsilon =5\%\right)}$ at the austenitizing temperature of 850 °C at the lowest annealing temperature (Q2-180 °C). This maximum corresponds to the increase of hardness with increasing dissolved carbon content, since the dissolvability of carbon and chromium is increasing with increasing austenitizing temperatures. Above *T*_A_ = 850 °C, the increasing dissolvability of carbon causes the martensite finish temperature to fall below room temperature which results in a higher retained austenite content. The increasing austenitizing temperature of course also involves a decrease in the amount of carbides. These effects and the aforementioned (in Section 2.1) grain growth with increasing austenitizing temperature therefore lead to a decrease of hardness as well as strength of the material. The main effect of annealing is the relaxation of residual stresses due to diffusion of atoms, motion of dislocations and voids, as well as the formation of carbides. At low annealing temperatures, the hardness only slightly decreases as the tetragonal martensite transforms into cubic martensite. With higher annealing temperatures, the precipitation of finely-distributed carbides is enhanced due to higher diffusion rates, resulting in reduced hardness and higher ductility. There is an opposed effect at higher austenitizing temperatures, since for hardened conditions, with higher retained austenite contents, the transformation of retained austenite to martensite increases the hardness with increasing annealing temperatures (e.g., Q3-230 °C and Q4-230 °C) until the relaxation effects of carbide formation predominate.

Regarding the expected mechanical properties of the different heat treatment conditions of 100Cr6, the trends of tensile strength values *R*_m_ out of conventional tensile tests (Figure 2) are considered as a validation approach of the compression test on spherical micro samples. Note that, since the stress value σ of compression tests is only a descriptor, absolute values should not be compared to those of standardized tensile tests. Due to superposition of different effects during heat treatment of 100Cr6, the development of the stress descriptor σ is similar to the resulting trend of the tensile strength in dependence of austenitizing and annealing temperatures in Figure 2. Although the amount of experimental data points of the descriptor of compression tests is not sufficient to replicate the whole material data of 100Cr6, as in [19], the analog trend is still discernible. The dashed line of Figure 2 is recreated by using the online tool WebPlotDigitizer (version 4.2, Ankit Rohatgi, San Francisco, CA, USA) [22] and is displayed as a black reference line in Figure 9. The interrelation between the stress descriptor of micro compression tests and the tensile strength seem to be not linear and shifted. Variations in the chemical composition of the examined alloys might explain the differences in the contour plots, as the carbon content of the tensile specimens was slightly lower (0.97 wt.%) [19]. Furthermore, the initial material condition before heat treatment (e.g., the amount of carbides) highly influences the resulting microstructure. However, transferability and scalability from a compression test on micro samples to a material property on the macro level seem possible.

Additional experiments are needed to further validate the trend of the mechanical material property of 100Cr6 in areas of special interest, for example, of material conditions with the expected highest values of the stress descriptor σ in the range of temperatures of *T*_A_ = 800–950 °C and *T*_T_ = 180–230 °C.

In general, during micro compression test on spherical samples, mechanical effects and material-specific influences superpose and affect resulting descriptor values. The mechanical effects include the contact mechanics of sphere and plane with point contact at the beginning of the test, complex stress development due to sample geometry, friction and surface roughness of contact partners. Influences like diffusion, motion of dislocations, residual stresses, local distribution of carbides, grain boundaries, stress-induced phase transformation and alloy dependencies belong to the material-specific effects occurring during mechanical loading. For a better understanding of these effects and the improvement of the interpretation of the extracted descriptors, a simulation of the micro compression test will be part of future research work.

## 5. Conclusions

In this work, the relevance of micro compression testing for the determination of mechanical descriptors in a short-term characterization method on spherical micro samples, as in the CRC 1232, was demonstrated.

The evaluation of descriptors extracted from force-displacement curves and calculated stress-strain relations proves the robustness of this testing method, because scatter and standard deviations are small. Due to the sample geometry, descriptors at higher load levels show less scattering and examined trends tend to be more pronounced than for lower load levels. Due to a greater deformation of the sample and a bigger loaded sample volume, small errors (e.g., due to surface roughness or deviations in geometry accuracy) can be compensated. In this study it is shown that the descriptors of the micro compression testing method are sensitive to heat treatment changes and different microstructures. However, the higher the load, the greater the possible detection of differences between different investigated materials. Nevertheless, it should be noted that there is a maximum limit of loading, since with greater deformations the geometry of the sphere transforms into a disc, resulting in higher frictional influences, thus affecting the results significantly.

Regarding the transferability of mechanical material characterization by compression test on microspheres to conventional material properties of macro material, a transfer is found to be promising, as the examined trend of a stress descriptor interrelates with the trend of tensile strength from literature. Of course, since there is yet only a small area investigated by micro compression tests, it is necessary to perform further investigations to determine a direct correlation between descriptors of a fast characterization technique on micro samples and a material property on a macro scale. This study can therefore be extended with experiments on additional heat treatments of 100Cr6 in areas of special interest (e.g., maximum descriptor values), but also with investigations of different alloy compositions. All in all, the micro compression test on spherical samples can then be used in high throughput-based screening processes for the development of new alloys, as in the CRC 1232, since it provides useful descriptors very fast compared to conventional material testing methods.

## Figures and Tables

**Table 1:** Chemical composition of the investigated microspheres in comparison with the limitations of the DIN EN ISO 683-17:2000-04 [20].

Material		Chemical Composition (wt.%)							
		Fe	C	Cr	Mn	Ni	P	S	Si
100Cr6 (AISI 52100)	−	bal.	1.07 ^2^	1.31 ^1^	0.35 ^1^	0.17 ^1^	−	0.018 ^2^	0.35 ^3^
DIN EN ISO 683-17:2000-04	min max	bal.	0.93 1.05	1.35 1.60	0.25 0.45	− 0.40	− 0.025	− 0.015	0.15 0.35

^1^ By atomic absorption spectrometry (AAS); ^2^ by combustion analysis; ^3^ by photo metric analysis.
